# Comparative genomic analysis of *Mycoplasma pneumoniae* isolated in the United Kingdom, between 2016 and 2024

**DOI:** 10.1186/s12864-025-12101-y

**Published:** 2025-10-08

**Authors:** Rediat Tewolde, Joshua C. D’Aeth, Rebecca Thombre, Michael L. Beeton, Richard S. Rowlands, Samuel K. Sheppard, Matthew D. Hitchings, Ben Pascoe, Victoria J. Chalker, Baharak Afshar

**Affiliations:** 1 Respiratory and Vaccine Preventable Bacteria Reference Unit (RVPBRU), UK Health Security Agency (UKHSA), London, UK; 2 Department of Biomedical Sciences, Microbiology and Infection Research Group, Cardiff Metropolitan University, Cardiff, UK; 3 Department of Biology, Ineos institute for Antimicrobial Research , University of Oxford, Oxford, UK; 4 Institute of Life Science, Swansea University Medical School, Swansea University, Swansea, UK; 5 NHS England, London, UK; 6 Faculty of Veterinary Medicine, Chiang Mai University, Muang, Chiang Mai Thailand; 7 School of Animal and Comparative Biomedical Sciences, University of Arizona, Tucson, AZ USA

**Keywords:** *Mycoplasma pneumoniae*, Whole genome sequencing (WGS), Population genomics, Antimicrobial resistance (AMR), Macrolide resistance

## Abstract

**Background:**

*Mycoplasma pneumoniae* is a bacterium that causes upper and lower respiratory infections in children and adults. Epidemics occur over the winter period every few years with the most recent seen in 2023/24. These epidemics are thought to be due to the circulation of p1 type variants of *M. pneumoniae* which have cyclic epidemiology, with the peaks of infection driven by naïve individuals, mainly those < 5-years-old. Currently there is no routine monitoring or genomic surveillance to detect variants of *M. pneumoniae* circulating in the UK. We analysed 38 genomes of *M. pneumoniae* isolated in the UK between 2016 and 2024 for the detection of Macrolide-resistant *M. pneumoniae* (MRMP) and typing of *M. pneumoniae* variants. Core-genome analysis was used to compare the *M. pneumoniae* UK data with the global genomic data of 290 *M. pneumoniae* strains from 14 countries.

**Results:**

Of the 38 isolates from UK, the majority were obtained from male patients (62%), with the most common age range (38%) being the 0–15 years age group. All 38 UK isolates were identified as *M. pneumoniae*, of which 19 isolates (50%) were identified as p1-type 1 strains, and 19 isolates (50%) as p1-type 2. Of 38 isolates, six (16%) isolates were MRMP, of which four (67%) MRMP isolates were identified as sequence type (ST)3 from 2024 and the remaining two MRMP isolates from 2016 to 2024 were identified as ST14. Two core-genome phylogenetic trees; one based on strains from UK only (38 strains) and the other on a combined dataset of global and UK strains (328 strains), revealed two main clades corresponding to p1 types (p1-type 1 and p1-type 2 clades). Within these clades, strains were generally grouped into subclades according to their sequence type (ST), with a few exceptions observed in the global dataset. The phylogenetic tree for the 328 strains (290 global data and 38 UK data) showed 7 Subclades: Subclade 1 (ST3), Subclade 2 (mainly ST3/ST20), Subclade 3 (ST17), Subclade 4 (ST1), Subclade 5 (mainly ST14), Subclade 6 (mainly ST7/ST47) and Subclade 7 (ST2). ST3 strains were positioned in two Subclades (Subclade 1 and Subclade 2). 99% of the isolates in Subclade 1 (ST3) were MRMP. ST3 MRMP includes the most predominant strains in East Asia (Taiwan, Korea, Japan and China). Subclade 1 (ST3) was predominant in the strains isolated in 2024 from the UK that were included in this study out of which three ST3 MRMP strains were clustered in Subclade 1. Macrolide resistance was present in all Subclades except Subclade 7 (ST2).

**Conclusions:**

It is imperative to continuously monitor Subclade 1 in the UK to better understand the burden of macrolide resistance and the potential spread and evolution of this strain within the population. Whole genome sequencing based genomic surveillance of *M. pneumoniae* is a vital tool for the detection of macrolide resistance, and the surveillance of circulating strains during outbreaks and seasonal epidemic surges.

**Supplementary Information:**

The online version contains supplementary material available at 10.1186/s12864-025-12101-y.

## Background

*Mycoplasma pneumoniae* (*M. pneumoniae*) belongs to the class Mollicutes and is a bacterium that causes upper and lower respiratory infections in children and adults [1]. It is the causative agent in 15–20% of community acquired pneumonia (CAP) cases in adults and up to 40% of cases in children [2, 3]. *M. pneumoniae* has a small genome, size of ≈ 800 kb with 700 coding operons and a slow generation time (6 h) [3]. *M. pneumoniae* infection symptoms are usually characterised by an unresolving persistent cough, low-grade fever, headache, hoarseness, and rarely (5% of cases) bullous myringitis (ear drum infection). Diseases caused by *M. pneumoniae* are generally self-limiting and limited to respiratory infections, however other extrapulmonary complications have also been observed. These include Stevens-Johnson syndrome, meningitis, encephalitis, haemolytic anaemia, myocarditis, pericarditis, and septic arthritis.

Genetically, *M. pneumoniae* can be classified into two groups which are based on the different variations in the repetitive elements RepMP2/3 and RepMP4 in the p1 protein gene *MPN 141* [4]. The p1 protein is a part of the adhesion complex and plays a role in adherence to the respiratory epithelium of the host cell, facilitating host cell and tissue disruption [3]. The two groups are designated Subtype 1 (p1-type 1) and Subtype 2 (p1-type 2) [5]. Many methods such as p1 typing, multilocus variable-number tandem-repeat analysis (MLVA), multilocus sequence typing (MLST) via Sanger sequencing, and whole genome sequencing (WGS), can be used for the typing of *M. pneumoniae* [6]. A switch between these two subtypes (p1-type1 and p1-type 2) is thought to be responsible for the periodic epidemic surges of *M. pneumoniae* [7].

*M. pneumoniae* epidemics occur during winter every 4–7 years globally [8]. The most recent increase in *M. pneumoniae* cases in the UK, and other countries, was observed in late 2023/24 [9]. There are many hypotheses that may be used to explain the recent delayed re-emergence in *M. pneumoniae* infections. Firstly, this may be due to non-pharmaceutical interventions against COVID-19 [9]. Secondly, it may be due to emergence of new variants or p1 subtypes of *M. pneumoniae*, to which the public does not have immunity. The cyclic epidemic nature of *M. pneumoniae* infections in the UK may result from the introduction of new variants into a population in which immunity is notpresent [10]. Macrolide-resistant *M. pneumoniae* (MRMP) is a growing concern. Data from China have shown that MRMP accounts for > 90% of cases and is associated with specific circulating variants [11]. In Europe, this value is closer to 10%, depending on the region and time of sampling [12].

Currently there is no routine monitoring or genomic surveillance of the p1 variants of *M. pneumoniae* circulating in the UK. Among the typing methods available, p1 typing helps in determination of strain variants [13], MLVA offers information on strain differentiation [14], and MLST offers details on sequence types (STs) [15]. Ideally, all three methods should be used for comprehensive surveillance, but this is time consuming, labour intensive and not cost effective. Whole genome sequencing (WGS) can bridge the gap between these typing methods, while incorporating additional detail on genetic diversity, p1 types, sequence types, macrolide resistance and potential virulence markers of *M. pneumoniae* [16].

In the present study, we analysed 38 genomes of *M. pneumoniae* isolated in the UK between 2016 and 2024 using WGS for the detection of MRMP and typing of *M. pneumoniae* variants. Furthermore, this study aimed to use core-genome analysis to compare the *M. pneumoniae* UK data with the global genomic data of 290 *M. pneumoniae* strains from 14 countries, isolated between 1944 and 2024.

## Methods

### Laboratory analysis

#### Clinical specimens

Respiratory samples were submitted to the Zoonotic and Acute Respiratory Section, Respiratory and Vaccine Preventable Bacteria Reference Unit (RVPBRU), UK Health Security Agency (UKHSA), for *M. pneumoniae* confirmation using an in-house qPCR assay and macrolide resistance testing. The RVPBRU provides a *M. pneumoniae* primary diagnostic service and a service for confirmation of infection on samples tested positive locally and for identification of presumptive *M. pneumoniae* isolates. The clinical samples received include sputum, throat swabs, endotracheal aspirates, nasopharyngeal aspirates and nose and throat combined swabs. *M. pneumoniae* is a non-notifiable organism in England and Wales, and therefore data is reported on a voluntary basis, and only positive results are reported. 

#### Sample processing for DNA extraction and qPCR testing

The clinical samples were processed using the KingFisher™ Duo Prime DNA extraction platform (Thermo Scientific™) followed by performing an in-house real-time qPCR assay based on the detection of the *M. pneumoniae* p1 adhesion gene. Samples that were *M. pneumoniae* qPCR positive (with a Ct value < 30) and sent in non-VTM (viral transport medium) swabs were cultured and culture positive samples were tested using WGS method.

#### Macrolide resistance testing

Samples that were *M. pneumoniae* qPCR positive were subjected to macrolide resistance testing using a block-based PCR and Sanger sequencing. BioNumerics software (https://www.bionumerics.com/) was used to detect the presence of known point mutations in domain V of 23S rRNA conferring macrolide resistance (MR) to *M. pneumoniae.* Macrolide resistance was also determined by WGS. (Supplementary Table 1: Patient and Genomic Data for 38 *M. pneumoniae* isolates from UK).

#### M. pneumoniae culture preparation

The clinical samples that were *M. pneumoniae* qPCR positive were initially cultured in 2mL aliquot of Mycoplasma broth (Mycoplasma Experience Ltd, UK) and Mycoplasma agar (Mycoplasma Experience Ltd, UK). All broths were incubated at 37 *±* 2 °C and plates were incubated at 37 *±* 2 °C in ~ 10% CO_2_/6% O_2_ (generated using a CampyGen system microaerophilic GasPak kit) in an anaerobic jar for up to 6 weeks or until growth was observed. To increase the genomic DNA yield for WGS, isolates were Sub-cultured in 30–100 mL Mycoplasma broth for 2–6 weeks until growth was observed. Broth cultures were then centrifuged at 20,000 g for 10 min and the pellet obtained was used for DNA extraction using the QIAsymphony DSP Virus/Pathogen Midi Kit (Qiagen) at UKHSA, and a manual QIAamp DNA extraction kit (Qiagen) was used at Cardiff Metropolitan University.

#### Whole genome sequencing

In total, 38 *M. pneumoniae* isolates collected in the UK, between 2016 and 2024 were sequenced in this study. Several of these isolates archived at the UKHSA reference laboratory (RVPBRU; 25/38), which were isolated between 2016 and 2020 were previously sequenced using an Illumina MiSeq 150 bp read size. More recent isolates, including those isolated from the recent upsurge (in 2024) were sequenced using an Illumina NextSeq 1000 100 bp read size. The reads (FASTQ files) were trimmed for quality using Trimmomatic (version 0.39) [17] to remove bases with a quality score (PHRED) below 30 from both ends.

### Genomic analysis

#### Species identification

A K-mer (a short string of DNA of length k) based approach was used (https://github.com/phe-bioinformatics/kmerid*)* to confirm the identity of the sample and to ensure that the sequence was free from contamination and was not a mixed culture.

#### MLST

MLST analysis was performed to identify genetically related isolates by sequence type (ST) using Metric Orientated Sequence Typer (MOST) available from https://github.com/phe-bioinformatics/MOST [18]. MLST was undertaken using the MLST database https://pubmlst.org/bigsdb? db=pubmlst_mpneumoniae_seqdef described by Brown et al., 2015 [15].

#### p1 typing

For the subtyping of *M. pneumoniae* isolates, the *MPN141* (p1) and *MPN528a* genes were examined as described previously [19]. In the genomic sequence of *M. pneumoniae* strain M129, the *MPN141* gene has the nucleotide T in p1-type 1 strains and a C in p1-type 2 strains at position 184,991; the *MPN528a* gene has the nucleotide A in p1-type 1 strains and a C in p1-type 2 strains at position 650,584 [19]. GeneFinder software (version 2–7) (https://github.com/ukhsa-collaboration/gene_finder) was used to identify variants in the *MPN141* and *MPN528a* genes.

#### Antimicrobial resistance (AMR)

Macrolide resistance in *M. pneumoniae* is mediated via a single point mutation in the 23S rRNA gene within the 50 S ribosomal subunit. The mutation is typically found in the positions A2063G, A2063C, A2064G, A2064C, A2067G, C2617G or C2617A. GeneFinder software was used to detect point mutations at 23 S rRNA resistance locus associated with MRMP.

#### Virulence genes

The nucleotide sequences of Community-Acquired Respiratory Distress Syndrome (CARDS) toxin (MPN372), hmw1 (MPN447), hmw2 (MPN372) and hmw3 (MPN452) from *M. pneumoniae* M129 were download from the Virulence Factors of Pathogenic Bacteria (VFDB) database (http://www.mgc.ac.cn/cgi-bin/VFs/genus.cgi?Genus=Mycoplasma) and used as reference genomes for the determination of virulence genes. GeneFinder software was used to determine variants found in virulence genes.

### Phylogenetic analysis

#### Core-genome SNP-based phylogenetic analysis using UK isolates

For the 38 *M. pneumoniae* UK isolates, single nucleotide polymorphisms (SNPs) were determined via the in-house UKHSA SNP pipeline PHEnix tool (https://github.com/ukhsa-collaboration/PHEnix*)* on the *M. pneumoniae* isolates against the reference *M. pneumoniae* M129 genome (GenBank accession no. NC_000912.1, a macrolide-susceptible Sequence Type (ST) 1 strain isolated in the United States during 1968). The PHEnix pipeline produced VCF files and an alignment. This alignment was then used as input for Gubbins (version 3.4) https://sanger-pathogens.github.io/gubbins to remove recombination events from the alignment and produce a phylogeny using RAxML (version 8.2.12) https://cme.h-its.org/exelixis/software.html. The Snp-dists tool (https://github.com/tseemann/snp-dists) was then used to calculate the SNP distances from the alignment that Gubbins pruned of recombination events. The resulting phylogenetic tree was visualized using iTOL (version 3.1) https://itol.embl.de/help.cgi#anno, which was also used to overlay metadata information. To visualise genomic information such as recombination blocks, the resulting phylogenetic tree were also visualized by using Phandango (version 1.3.0) https://jameshadfield.github.io/phandango/#/.

#### Genome assembly and core-genome K-mer-based phylogenetic analysis using global isolates

The UK sequences were compared to 302 sequences (280 assemblies and 22 fastq files) of *M. pneumoniae* strains isolated globally.

The global *M. pneumoniae* genome sequences were downloaded from the European Nucleotide Archive (ENA) database on 17th October 2024 (Supplementary Table 2: Patient and Genomic Data for 290 *M. pneumoniae* isolates obtained from the NCBI RefSeq database). A total of 22 fastq files were assembled using Spades *de novo* assembly software [20] with the following parameters ‘spades.py–careful − 1 strain.1.fastq.gz −2 strain.2. fastq-t 2-k 21,33,55,77. All the contigs were checked for quality. Contigs of less than 500 bp were removed from the assemblies and QUAST8 (version 5.2.0) was used for quality control [21]. A total of 12 isolates (including one ST3 MRMP isolate collected in UK in 2024) did not meet the standard *M. pneumoniae* genome length (653000-980000 bp based on the NCBI guide) and one isolate with N50 < = 5000 was removed.

PopPUNK (version 2.5.0) [22] was used to split the combined 328 (290 Global data and 38 UK data) assemblies into unique strains based on core and accessory genome distances. PopPUNK was used to create a core-genome distance phylogeny for the final collection of sequences. The Neighbour-joining phylogenetic tree was visualised using iTOL (version 3.1) (https://itol.embl.de/help.cgi#anno).

We performed additional phylogenetic analysis on the isolates within Subclade 1 of the Global phylogeny. The assemblies of these isolates were mapped to the complete chromosome of *M. pneumoniae* strain S355 (Accession code: CP013829) using ska v1.0 (https://github.com/simonrharris/SKA). Gubbins v3.4, with the RAxML tree builder, was then used to create a recombination-corrected phylogeny for the subclade.

### Data deposition

The sequence data for the 38 genomes of *M. pneumoniae* sequenced in this study were deposited in the SRA database (accession no. PRJNA1231462).

## Results

### *M. pneumoniae* isolates from the UK

A total of 38 *M. pneumoniae* strains were tested in this study. These isolates were from pre and post COVID-19 pandemic period (2016–2020 and 2024); including 4 isolates from 2016, 2 isolates from 2017, 3 isolates from 2018, 2 isolates from 2019, 13 isolates from 2020, and 13 isolates from 2024 with 1 positive control (*M. pneumoniae* strain NCTC 5167). (Supplementary Table 1: Patient and Genomic Data for 38 *M. pneumoniae* isolates from UK).

Patient details like patient age and patient gender were available from the UKHSA laboratory information management system (LIMS) database (Supplementary Table 1: Patient and Genomic Data for 38 *M. pneumoniae* isolates from UK). The age distribution was as follows: 14 patients (38%) were aged 0–15 years, 9 patients (24%) were aged 16–35 years, 11 patients (30%) were aged 36–65 years, and 3 patients (8%) were aged > 66 years. 14 patients (38%) were female, and 23 patients (62%) were male. (Supplementary Table 1: Patient and Genomic Data for 38 *M. pneumoniae* isolates from UK).

### Genomics of UK isolates

Strain characteristics: All 38 UK isolates were identified as *M. pneumoniae* using the KmerID software, of which 19 isolates (50% of collection) were p1-type 1 strains, and 19 isolates (50%) were p1-type 2. Six (16%) isolates were MRMP. Five of the MRMP isolates (1616466 (ST3, p1-type 1), 1616469 (ST3, p1-type 1), 1616470 (ST3, p1-type 1), 1616474 (ST3, p1-type 1), and 1616468 (ST14, p1-type 2)) had the point mutation A2063G and one of the macrolide resistant strains (76_S62_L001(ST14, p1-type 2)) had the point mutation A2064G in the 23S rRNA gene (Supplementary Table 1: Patient and Genomic Data for 38 *M. pneumoniae* isolates from UK). Among the six MRMP isolates, four (67%) isolates were identified as ST3 from 2024. SNPs conferring macrolide resistance in the six MRMP isolates were also identified by Sanger sequencing, consistent with the WGS results (Supplementary Table 1: Patient and Genomic Data for 38 *M. pneumoniae* isolates from UK).

Gene specific analysis: The important virulence factor Community-acquired respiratory distress syndrome toxin (CARDS TX), was found in all 38 UK isolates (100%). Within CARDS TX, we identified one non-synonymous SNP (T1112G, I371S) in all p1-type 2 strains but no variation in any p1-type 1 strains (Supplementary Table 1: Patient and Genomic Data for 38 *M. pneumoniae* isolates from UK).

The high-molecular-weight proteins HMW1, HMW2, and HMW3 are required for cytoadherence [23, 24]. We identified the G173A: T241C mutation in all p1-type 2 strains in the HMW1 gene and the T88C mutation in all p1-type 2 strains in the HMW2 gene. No mutation was detected in the HMW3 gene in either p1-type, except for the C587T mutation in ST2 (p1-type 2) (Supplementary Table 1: Patient and Genomic Data for 38 *M. pneumoniae* isolates from UK).

**Fig. 1:**
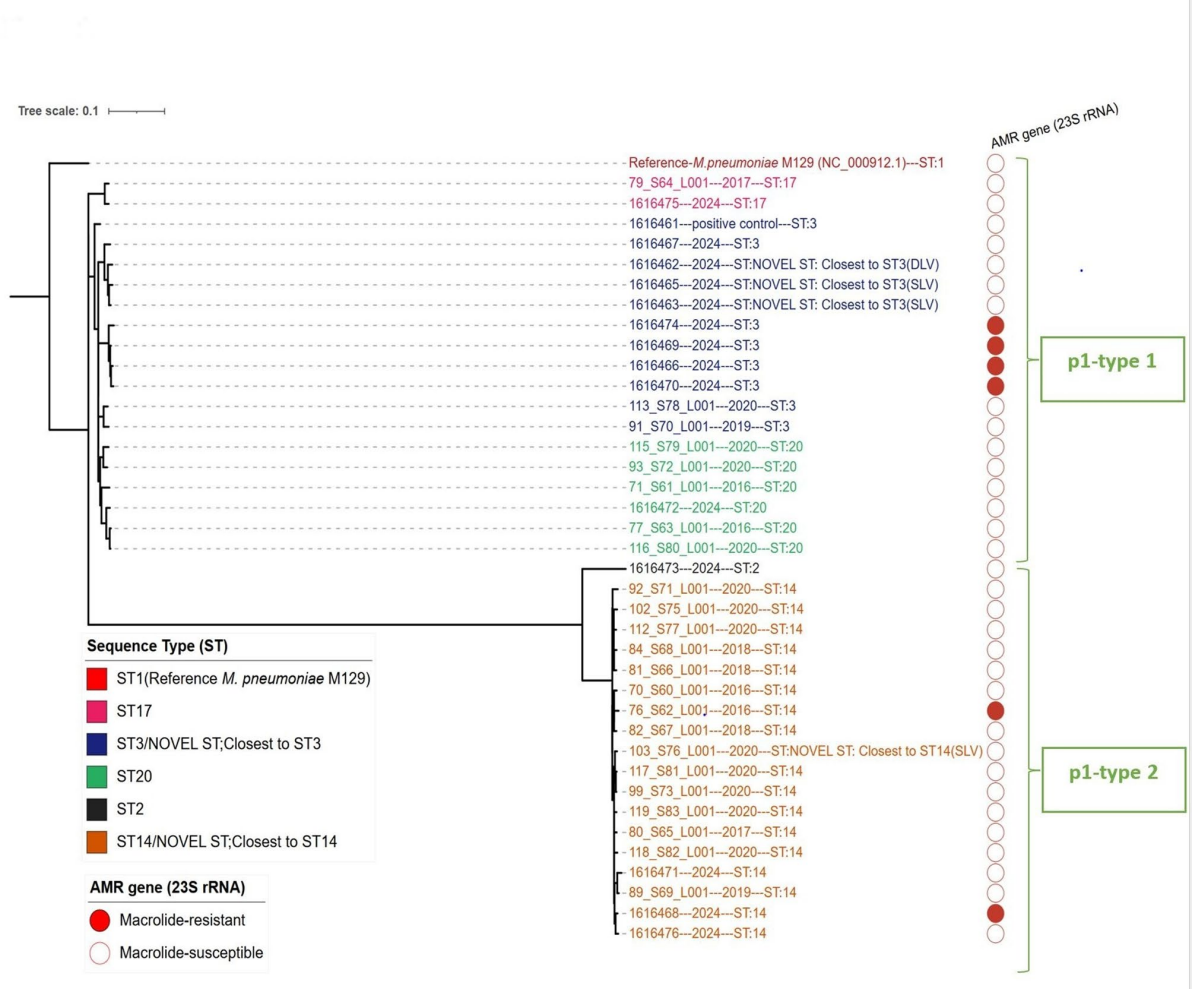
Maximum likelihood phylogeny created from single nucleotide polymorphisms (SNPs) using 38 *M. pneumoniae *genomes (from 37 isolates from the UK and a positive control *M. pneumoniae* NCTC 5167) from 2016-2024. Variants were called by using the Reference-*M. pneumoniae* M129 genome (a macrolide-susceptible Sequence Type 1 strain isolated in the United States during 1968 (GenBank accession no. NC_000912.1)). Tips are labelled by isolate ID—Date of Isolation---ST. Coloured strips (red filled circles) indicate isolates with macrolide resistance-associated mutations. Tips are coloured by sequence type (ST). Data were visualized using iTOL 6.9.1 (https://itol.embl.de)

Core-genome SNP-based phylogenetic analysis using UK isolates: Phylogenetic analysis via core genome SNPs revealed two distinct clades according to p1 typing (Fig. 1: Maximum Likelihood phylogeny from 38 *M. pneumoniae* genomes). The phylogenetic tree revealed five subclades linked to certain MLSTs (ST2, ST3, ST14, ST17, and ST20). Furthermore, p1-type 1 UK clade (p1-type 1 strains) consisted of ST3, ST17, and ST20 isolates, and p1-type 2 UK clade (p1-type 2 strains) consisted of ST2 and ST14 isolates (Fig. 1: Maximum likelihood phylogeny based on single nucleotide polymorphisms (SNPs) from 38 *M. pneumoniae* genomes).

The phylogenetic tree shows that the four ST3 MRMP strains isolated in the UK in 2024 were in the same Subclade with an average of 13 SNPs distance within each other and at an average of 68 SNPs apart from other ST3 isolates (Fig. 1: Maximum likelihood phylogeny based on single nucleotide polymorphisms (SNPs) from 38 *M. pneumoniae* genomes). These four ST3 MRMP (from 2024) were widely geographically distributed, and the patients had no identifiable epidemiological links (Supplementary Table 1: Patient and Genomic Data for 38 *M. pneumoniae* isolates from UK).

### Recombination landscape

**Fig. 2:**
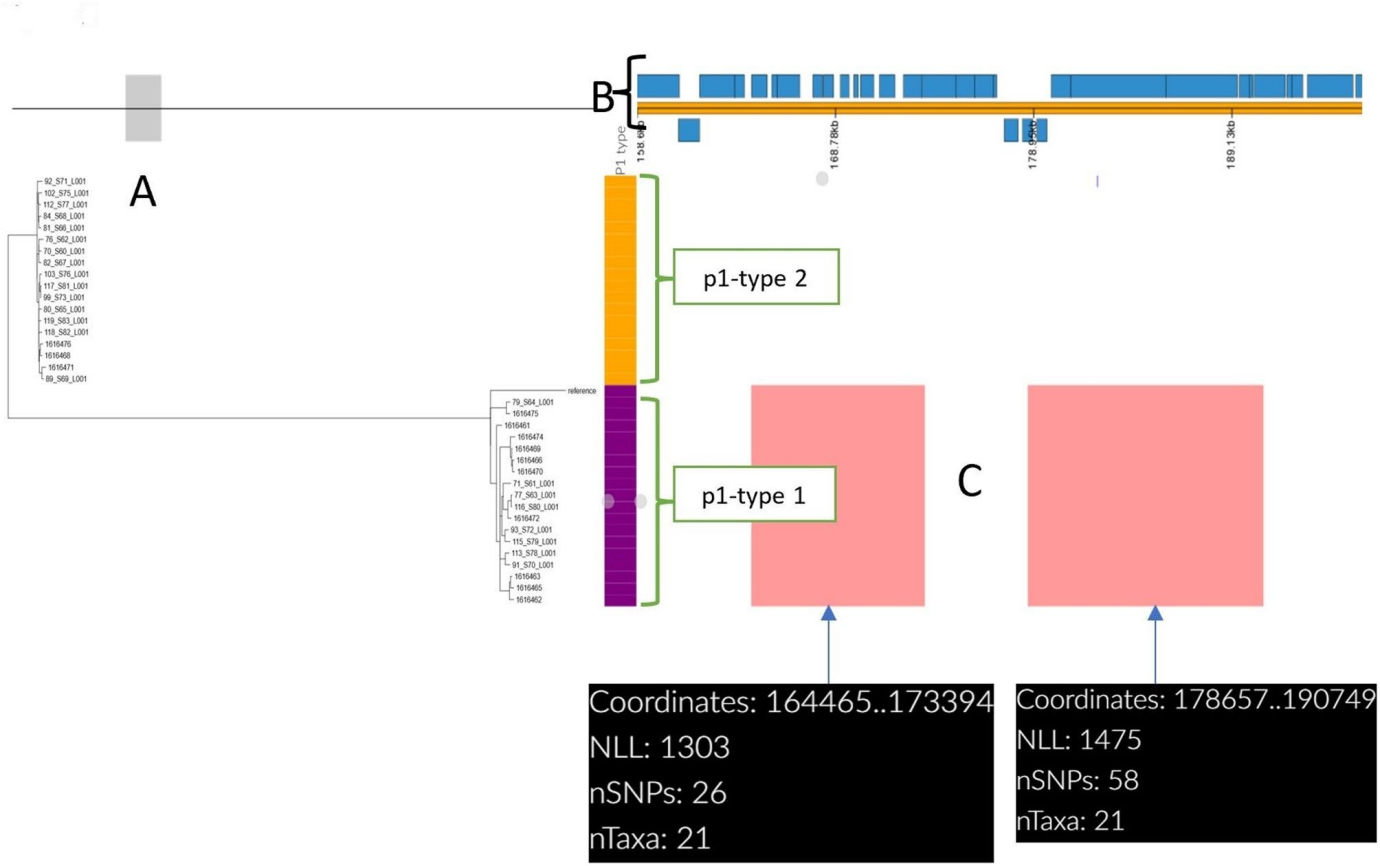
Displays the recombination regions identified from the 38 UK *M. pneumoniae* genomes. **A***M. pneumoniae* phylogeny based on single-nucleotide polymorphism for 38 *M. pneumoniae* genomes (from 37 isolates from the UK and a positive control *M. pneumoniae* NCTC 5167). **B** Annotated reference-*M. pneumoniae* M129 genome (a macrolide-susceptible Sequence Type 1 strain isolated in the United States during 1968 (GenBank accession no. NC_000912.1). **C** Coloured blocks represent putative recombination regions identified by using Gubbins software. Gubbins analysis identified 2 regions of predicted recombination (red lines) compared with Reference *M. pneumoniae* M129. The two coordinates (164465–173394; 178657–190749) of recombination sites were based on *M. pneumoniae* M129 strain. Data were visualized using Phandango (https://jameshadfield.github.io/phandango/#/)

By examining all 38 UK *M. pneumoniae* genomes in this study, we detected two recombination blocks in all the p1-type 1 clade (p1-type 1 strains) (Fig. 2). The first 8.9 Kb region, found at 164,465–173,394 bp in the reference, contained an adhesin p1 family protein, a DUF16 domain-containing protein, a thermonuclease family protein, and an ABC transporter ATP-binding protein. The second 12.1 Kb region, found at 178,657–190,749 bp in the reference, contained a DUF16 domain-containing protein, adhesin p1 and the cytadherence protein MgpC (Fig. 2, Supplementary Table 3).

### Genomics of UK and global data

#### Strain characteristics

**Fig. 3:**
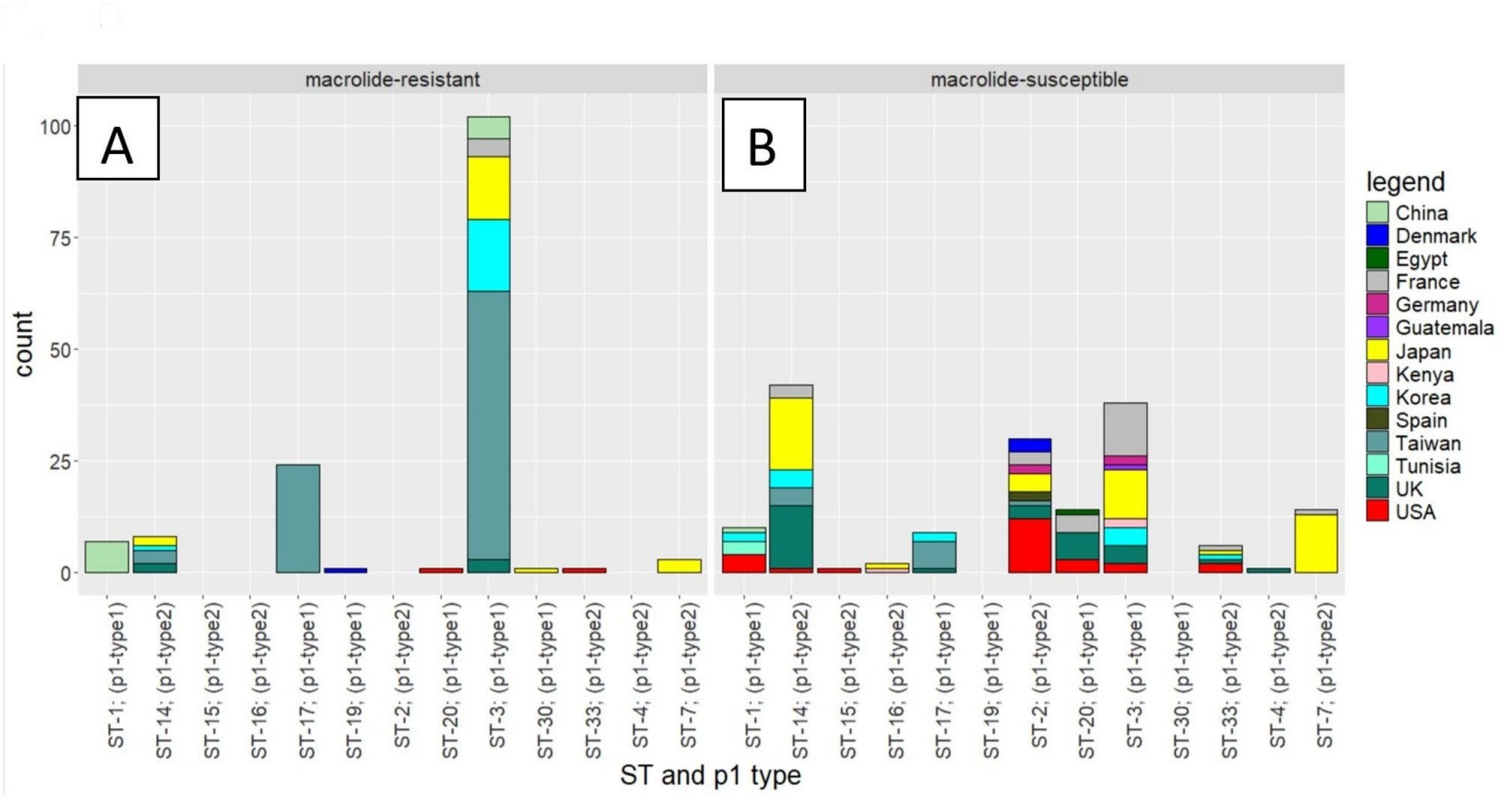
The relationship between macrolide susceptibility, p1 type, Sequence Type (ST) and country of isolation. Both plots are based on genomic data from 327 *M. pneumoniae* isolates obtained from the NCBI RefSeq database (global data) and UK data. These visualizations highlight the geographical diversity of *M. pneumoniae* macrolide-resistant and macrolide-susceptibility patterns. **A **The number of *M. pneumoniae *macrolide-resistant strains, categorized by sequence types (STs) and p1 types, across 14 counties. The y-axis represents the number of samples per ST, and each bar is color-coded based on the country of origin of the isolate. **B** The number of *M. pneumoniae* macrolide-susceptible strains, similarly, categorized by sequence types (STs) and p1 types, across the same 14 counties. The y-axis indicates the number of samples per ST, with bars color-coded by the country of origin

Incorporating an additional 290 publicly available *M. pneumoniae* genomes, 328 (290 Global data and 38 UK data) global genomes from *M. pneumoniae* strains isolated between 1944 and 2024, from 14 countries were analysed. The predominant global *M. pneumoniae* strains were identified as ST3 (*n* = 141/328; 43%) and ST14 (*n* = 49/328; 15%) (Supplementary Table 1: Patient and Genomic Data for 38 *M. pneumoniae* isolates from UK and Supplementary Table 2: Patient and Genomic Data for 290 *M. pneumoniae* isolates obtained from the NCBI RefSeq database). The AMR data showed that 152/328 (46%) of the strains were resistant to macrolides, with mutations in the 23S rRNA gene identified as follows: A2063G (131 strains), A2063T (13 strains), A2063C (2 strains), A2064G (3 strains), A2064C (1 strain), and C2617G (2 strains). MRMP was found most-commonly in *M. pneumoniae* ST3 strains (*n* = 107/328; 33%) (Supplementary Table 1: Patient and Genomic Data for 38 *M. pneumoniae* isolates from UK and Supplementary Table 2: Patient and Genomic Data for 290 *M. pneumoniae* isolates obtained from the NCBI RefSeq database, Fig. 3: Relationship between macrolide susceptibility, sequence type (ST), p1 type, and country of isolation).

#### Core-genome K-mer-based phylogenetic analysis using global isolates

**Fig. 4:**
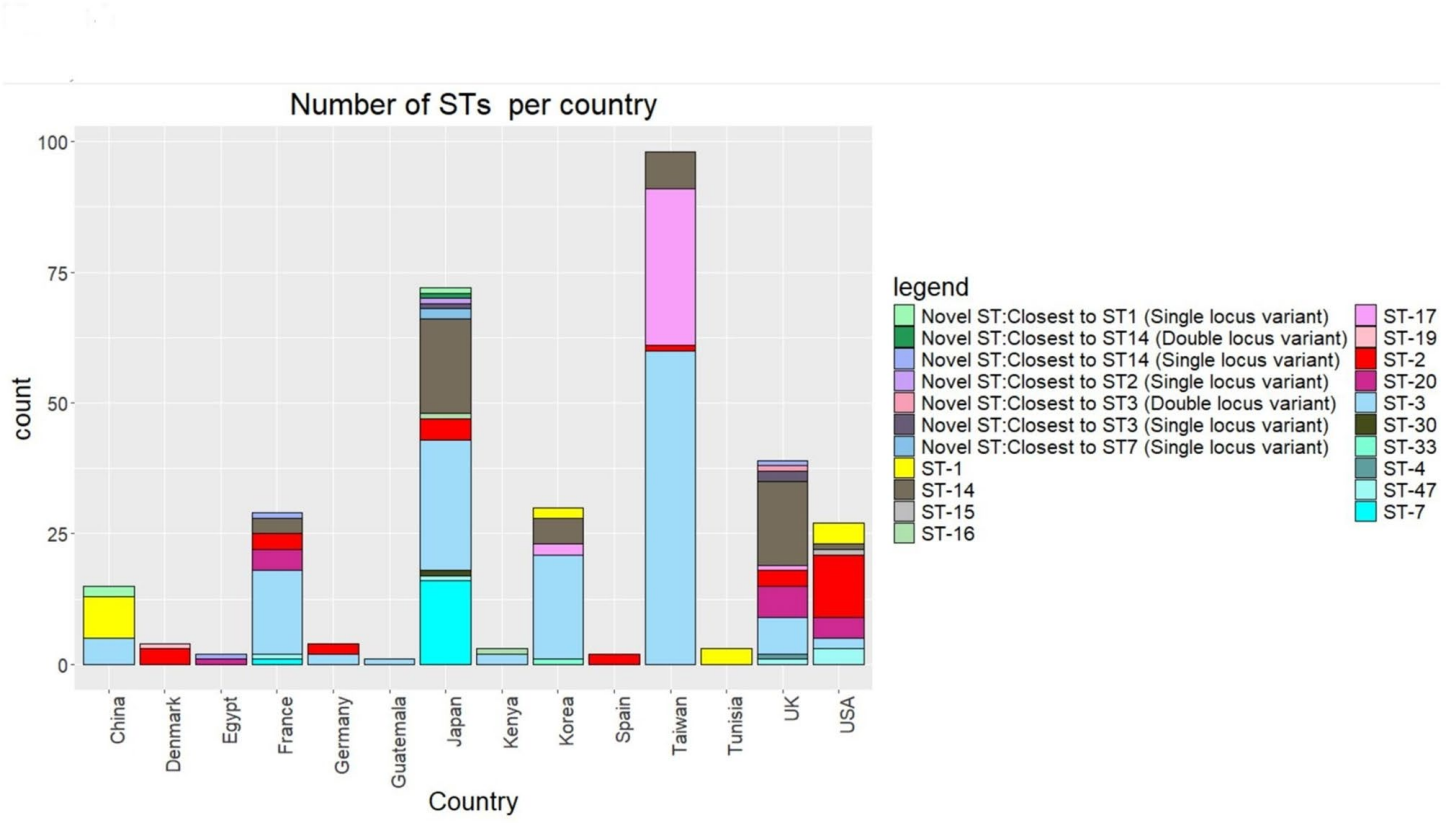
The number of *M. pneumoniae* sequence types (STs) categorized by country. The y-axis represents the number of samples per country, while each bar is color-coded based on the STs present within the isolates. The data includes genomic information from 327 *M. pneumoniae* isolates obtained from the NCBI RefSeq database, supplemented with data from the UK

**Fig. 5:**
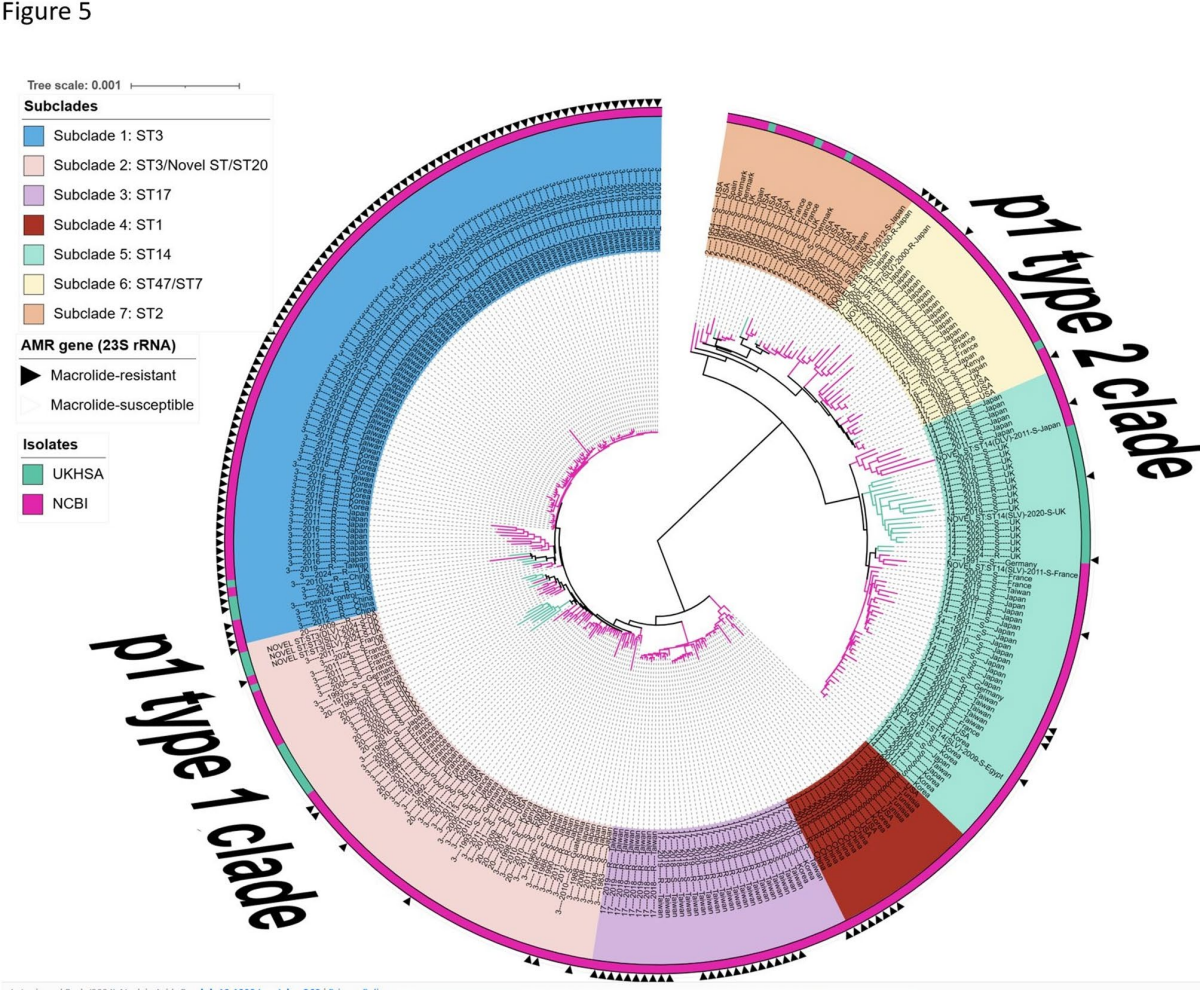
Neighbour-joining phylogeny formed from the core-genome distances estimated by PopPUNK on a collection 327 of NCBI RefSeq *M. pneumoniae *isolates from 14 countries (1944 to 2024). Arrows indicate MRMP isolates, outer annotation ring colours represent the Isolate origin, while the inner ring represents the Subclade designation in the tree. Branches of the tree are also coloured by the isolate origin (UKHSA or NCBI data)

To determine how the *M. pneumoniae* isolates are related globally, a phylogenetic tree was generated from 328 (290 Global data and 38 UK data) global genomes from *M. pneumoniae* strains isolated between 1944 and 2024, from 14 countries (Fig. 4: Distribution of *M. pneumoniae* sequence types (STs) categorized by country). The phylogenetic tree was divided into two major clades in accordance with p1 typing (p1-type 1 Clade and p1-type 2 Clade) (Fig. 5: Neighbour-joining phylogenetic tree based on a collection of *M. pneumoniae* isolates from UK and NCBI RefSeq database). Among the 328 strains identified, 216 (66%) were p1-type 1 and 112 (34%) was p1-type 2. The p1-type 1 clade was divided into four subclades (Subclade 1: ST3; Subclade 2: ST3/ST20; Subclade 3: ST17 and Subclade 4: ST1) and the p1-type 2 Clade was divided into another three subclades (Subclade 5: ST14/ST2; Subclade 6: ST47/ST7 and Subclade 7: ST2).

**Fig. 6:**
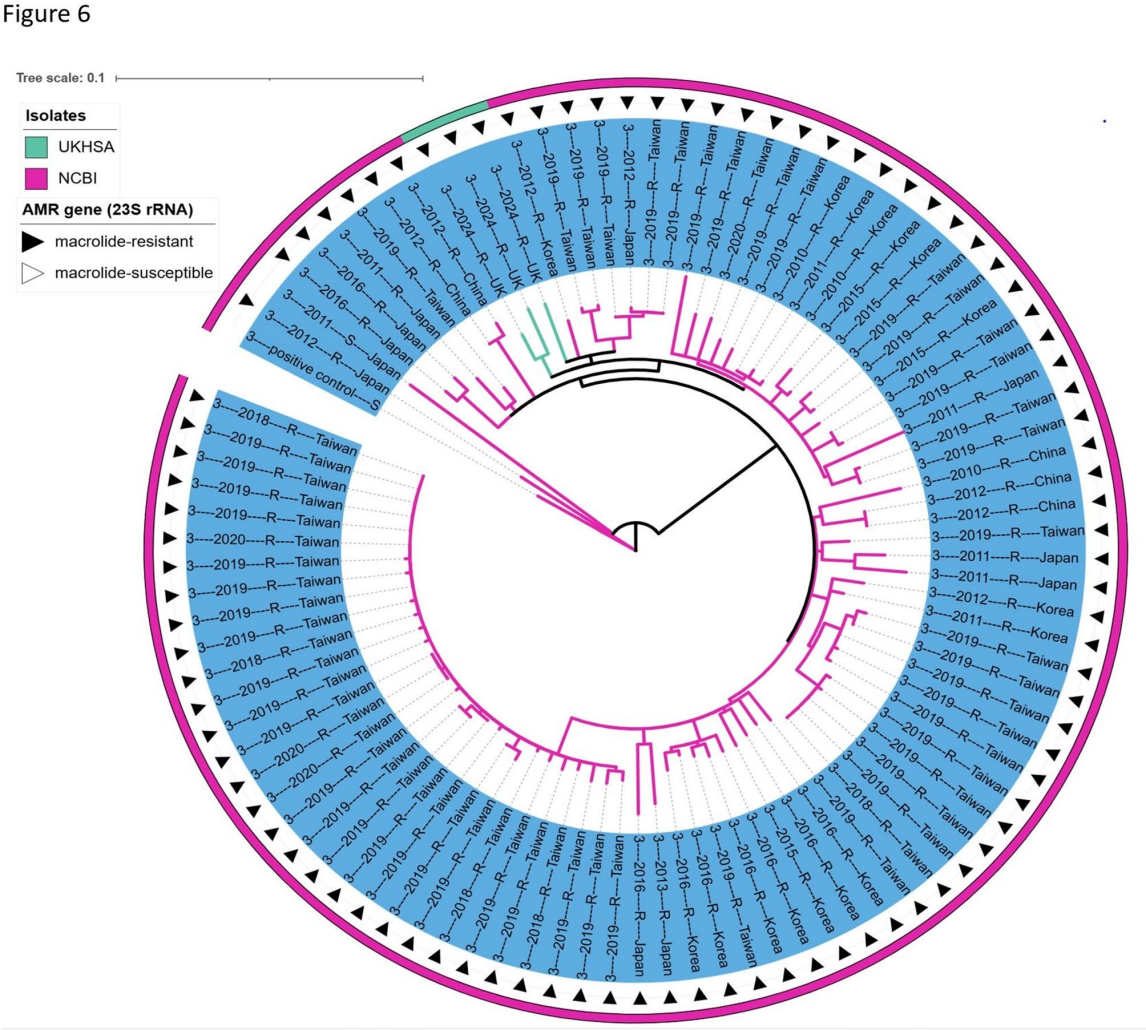
Phylogenetic tree of Subclade 1 (ST3), constructed from a global collection of *M. pneumoniae* isolates. The tree is labelled based on isolate origin and AMR gene, arrows indicate MRMP isolates, outer annotation ring colours represent the Isolate origin, while the inner ring represents the Subclade designation (Subclade 1) in the tree. Subclade 1 (ST3) represents the group of *M. pneumoniae* isolates across Taiwan, Japan, Korea, China, and the UK between 2010 and 2024, with 99% identified as macrolide-resistant *M. pneumoniae* (MRMP). This Subclade includes the most actively evolving strains, particularly in Taiwan. Notably, ST3 strains were also observed circulating in the UK in 2024

The ST3 strains were divided into two Subclades (Subclade 1 and Subclade 2). Subclade 1 (ST3) formed the largest subclade and had the highest number of MRMP (99%) strains. Further phylogenetic analysis of Subclade 1 revealed three MRMP ST3 UK strains clustered with the ST3 MRMP circulating in East-Asia (Subclade 1) (Fig. 5: Neighbour-joining phylogenetic tree based on a collection of *M. pneumoniae* isolates from UK and NCBI RefSeq database and Fig. 6: Phylogenetic tree of Subclade 1 (ST3), constructed from a global collection of *M. pneumoniae* isolates).

## Discussion

The present work was undertaken to use WGS to detect MRMP, p1 type, MLST, as well as to perform core-genome analysis. The study was run on a group of 38 *M. pneumoniae* strains isolated in the UK during 2016–2024. The strains were selected randomly to assess the viability of the WGS pipeline and to test the efficiency of the method. Core-genome analysis was used to compare the 38 *M. pneumoniae* UK data with the global genomic data of 290 *M. pneumoniae* strains from 14 countries isolated between 1944 and 2024.

*M. pneumoniae* is the primary cause of up to 40% of community-acquired pneumonia (CAP) cases in children [25]. *M. pneumoniae* epidemics occur every 3–7 years, however the major drivers responsible for the *M. pneumoniae* upsurges and the factors that influence transmission dynamics are still not understood [6]. A previous study reported a shift in the p1 subtypes during epidemic outbreaks of *M. pneumoniae* and suggested that a shift in the p1-type 1 and p1-type 2 subtypes may be a plausible driver of these *M. pneumoniae* outbreaks [26]. The delayed re-emergence of the *M. pneumoniae* epidemic was observed globally in 2024 [9], while the appearance of macrolide resistance, especially in China, during this period is a significant concern. Using metagenomic analysis, Li et al., 2024 identified 179 *M. pneumoniae* samples from East China, which were grouped into two epidemic clones: p1-type 1 (clade EC1) and p1-type 2 (clade EC2) [27]. While the EC1 (p1-type 1) clone is responsible for the elevated frequencies of macrolide-resistant *M. pneumoniae* (MRMP), the emerging EC2 (p1-type 2) clade now exhibits 100% macrolide resistance in East China [27].

Macrolides are the first line of treatment for infections caused by *M. pneumoniae* in many countries including the UK, and vaccine development, although still underway, has substantial hurdles. Hence, studying the presence of p1 subtypes and macrolide resistance in *M*. *pneumoniae* is crucial to further our understanding of seasonal epidemics.

Currently in the United Kingdom, there is no single surveillance system that fully captures national changes in *M. pneumoniae* activity. UKHSA has a multipartite and comprehensive respiratory surveillance program incorporating a wide range of community, syndromic and laboratory surveillance systems for monitoring known seasonal pathogens each winter [28]. An increase in the number of emergency department visits of patients with a diagnosis of pneumonia, especially those with *M. pneumoniae* infections was observed in England in the winter of 2023/2024 [28]. UKHSA’s voluntary surveillance database Second Generation Surveillance System (SGSS) is used to compile and present data related to *M. pneumoniae* laboratory detection. As infections caused by *M. pneumoniae* are not notifiable, this affects the surveillance data. Furthermore, only positive results are reported voluntarily, which may lead an underestimate of the number of cases or occurrence of disease caused by *M. pneumoniae*. Moreover, this is complicated by the fact that asymptomatic carriage, particularly in children can be missed during diagnosis.

In the UK, the methods for laboratory detection are based on serological detection of IgM or IgG levels in serum or detection of *M*. *pneumoniae* nucleic acid using nucleic acid amplification testing methods like PCR from clinical samples like throat swabs, nose or nasal swabs, bronchial aspirates, bronchoalveolar lavage, alveolar, nasopharyngeal aspirate, endotracheal aspirate, trachea, or sputum. However, these primary methods of laboratory detection do not report the presence of p1 subtypes, STs and macrolide resistance. A comprehensive surveillance typing method that can provide information on the p1 type, and the sequence types (STs) of the *M*. *pneumoniae* isolated during the epidemics is essential. This helps us understand the various p1 types that are circulating in the population and helps us gain a greater understanding of the STs circulating globally. Whole genome sequencing can help provide information on p1 typing, MLST profile, and can also help us understand the diversity in the p1 region.

It was observed that ST3 accounts for 43% (*n* = 141/328) of the *M. pneumoniae* strains present in the global data. This ST has been identified in China, France, Germany, UK, Korea, Taiwan, Japan, and the USA. The incidence of MRMP infections has increased worldwide, particularly in the Western Pacific region [29]. In Taiwan, a study by Hung et al. reported a Substantial increase in macrolide resistance rates from 12 to 24% during 2011–2016 to 54–88% during 2017–2020 [30]. A Substantial increase in macrolide resistance rates was also observed in the UK. In January and February 2024, macrolide resistance was identified in 5.2% (16 out of 309) and 4.7% (13 out of 276) of the samples, respectively; throughout the entire 2019–2020 season, resistance was detected in less than 1% (1 out of 110) of the samples (UKHSA 2024 *M. pneumoniae* surveillance report) [31]. In South China, 88.10% (*n* = 148) of the isolates from 2021 to 2023 were MRMP. This study also identified a high level of MRMP; 152/328 (46%) globally. For example, in South China, 100% (101 of 101) of the ST3 isolates from 2021 to 2023 were MRMP [32]. In the present study, we observed that MRMP is the most common in ST3 strains and is prevalent in Taiwan, Korea, Japan, China and the UK. The second most predominant MRMP sequence type was ST17 which was prevalent among strains in Taiwan. Of the 38 UK isolates in this study, 6 (16%) were macrolide resistant. Clinicians should be aware of macrolide resistant *M. pneumoniae* infection, both on initial and acquired infection. Genomic analysis indicated clonal expansion of the Lineage was Likely to have occurred. Whether this was with strains circulating in the UK or via importation cannot be determined with the current dataset and without improved genomic and epidemiological Surveillance. The travel history of the cases in this study was not known. Nonetheless, the presence of a novel sequence type in 2024 supports the theorem of naive population driving epidemics.

Core-genome phylogenetic analysis has been used to study genetic diversity in *M. pneumoniae* [16, 33, 34]. The two core-genome phylogenetic trees constructed and visualized in this study revealed notable findings (Fig. 1: Maximum likelihood phylogeny based on single nucleotide polymorphisms (SNPs) from 38 *M. pneumoniae* genomes, Fig. 5: Neighbour-joining phylogenetic tree based on a collection of *M. pneumoniae* isolates from UK and NCBI RefSeq database). First, the phylogenetic relatedness of the strains demonstrated a strong correlation according to p1 type. Second, each ST type was grouped by the same branch with some exceptions for the global data (Fig. 5: Neighbour-joining phylogenetic tree based on a collection of *M. pneumoniae* isolates from UK and NCBI RefSeq database). For example, within the global dataset, the ST3 strains were separated into two Subclades (Subclade 1 and Subclade 2). Subclade 1 (ST3) consists of the largest (99%) MRMP; including the most actively evolving strains in East Asia. Subclade 1 (ST3) was first detected in the UK in 2024 in this study, and the three ST3 MRMPs were clustered in Subclade 1 (Fig. 5: Neighbour-joining phylogenetic tree based on a collection of *M. pneumoniae* isolates from UK and NCBI RefSeq database and Fig. 6: Phylogenetic tree of Subclade 1 (ST3), constructed from a global collection of *M. pneumoniae* isolates). It was interesting to note the UK isolates situated within a clade of East Asian isolates in the recombination-corrected Subclade 1 phylogeny. This Suggests a successful international clonal expansion of this Lineage or multiple importation from other geographies. It is imperative to continuously monitor the spread and evolution of Subclade 1 in the UK, as well as in other countries, to monitor the further spread of this resistant lineage. WGS has proven to be a powerful tool in identifying this cluster, and its application should be expanded with global data from more isolates and countries to allow for further public health responses to threat antimicrobial resistance.

The present investigation identified the presence of CARDS TX in all *M. pneumoniae* isolates included in this study. The basic mechanism of CARDS TX is based on its property of cellular vacuolization and ADP-ribosyl transferase (ADPRT) activity. CARDS TX also causes cilia stagnation, nuclear fragmentation, and the release of inflammatory factors [35]. The presence of CARDS TX is related to the severity of disease [36] and it is significantly upregulated in humans during *M. pneumoniae* infection [37]. It is still unknown if variation in CARDS TX production within different *M. pneumoniae* strains affects disease severity [38]. We observed that all the p1-type 2 *M. pneumoniae* strains in the present study had a non-synonymous SNP (T1112G, I371S) which was absent in the p1-type 1 strains. (Supplementary Table 1: Patient and Genomic Data for 38 *M. pneumoniae* isolates from UK). A similar SNP was previously observed that differentiated the type 1 and type 2 strains [33]. It is not possible to elucidate if this SNP affected the activity of CARDS TX or affected the disease severity in the present study. But it is important to study this in future investigations to determine if variation in CARDS toxin in different strains and subtypes have any effect on strain virulence, persistence or severity in disease and clinical manifestations or if it may prove useful for immune-protection and vaccine design.

The high-molecular-weight proteins HMW1, HMW2, and HMW3 are essential for stability, adherence and gliding [39]. The internal structure of the surface adhesion complex of *M. pneumoniae* are made of terminal button that has HMW2, HMW3 and P65; bowl complex that has HMW2 and paired plates that is made of HMW1, HMW2, CpsG and HMW3 proteins [39]. In the present study it was observed that there was no mutation in HMW3 gene except for a unique C587T mutation in *M. pneumoniae* ST2 p1-type 2 strain. All the p1-type 2 *M. pneumoniae* ST 14 strains had two mutations G173A and T241C in HMW1 gene, while p1-type 2 *M. pneumoniae* ST 2 had only one mutation, T241C in HMW1 gene. All the p1-type 2 *M. pneumoniae* strains had a mutations T88C in HMW2 gene. There were no mutations seen in the HMW genes of the p1-type 1 *M. pneumoniae* strains. It is not possible to conclude if the mutation in HMW had any effect on the functioning or adhesion of *M. pneumoniae* p1-type 2 strains. Further research is warranted in this area to understand if mutations in these genes affects virulence adhesion or pathogenicity of different strains of *M. pneumoniae.*

Yu-Chia Hsieh et al., 2022 analysed 284 *M. pneumoniae* genomes for the investigation of recombination and found that the p1 adhesion gene is highly diverse and has multiple copies of the repeated regions RepMP4 and RepMP2/3 [34]. Different p1 variants/types are generated by DNA recombination between repetitive sequences (RepMP elements) in the *M. pneumoniae* genome [7]. Hence, we studied the putative recombination blocks present in *M. pneumoniae* isolates. This study detected putative recombination blocks covering an average of 2.3% of the genome, mainly in genes encoding for adhesin p1 family proteins and DUF16 domain-containing proteins (Supplementary Table 3: Sequence Coordinates of Putative Recombination Events). When performing core-genome SNP-based phylogenetic analysis, recombination regions were removed as recombination regions can have a variable effect on SNP analysis.

It is critical to recognize the limitations of this study. The sample size of the WGS study poses a significant drawback in terms of wider interpretation of the data. However, *M. pneumoniae* is difficult to culture. The organism can take up to 6 weeks to grow in broth culture and many clinical samples may test positive in the qPCR but are culture negative. Culture also depends on the type of sample, date of sample collection, type of transport medium used, and antibiotic treatment administered prior to sample collection. This difficulty in culturing isolates is also reflected in the relative paucity of publicly available *M. pneumoniae* genomes. Second, populations of *M. pneumoniae* can be found worldwide, but only genomes from 328 isolates were analysed in this study, which represented few countries and lacked global representation and geographical distribution.

The third limitation is that we did not study tetracycline-resistant *M. pneumoniae* (TRMP) or fluoroquinolone-resistant *M. pneumoniae* (FRMP). It is reasonable to assume that TRMPs and FRMPs may already be present in the genome, or that they are likely to evolve in the future, based on data from closely related *Mycoplasma* species. For example, fluoroquinolone-resistance is well documented in the closely related *Mycoplasma genitalium*, with dual resistance to macrolides and fluoroquinolones reported in greater than 50% of cases in parts of Asia [40]. Understanding the mechanisms of flouroquinolone resistance, and building a tool that could identify the likely point mutations related to TRMPs and FRMPs are vital for providing better guidance to inform clinicians of treatment options for MRMP isolates. To mitigate the challenges of *Mycoplasma* culture and reduce the turnaround time, culture-free NGS-based methods (e.g., NGS amplicon sequencing or target enrichment probe panels) or culture free metagenomics approaches could be used in the future for *M. pneumoniae* [41].

## Conclusion

In the present investigation, we conducted a study to explore the potential application of WGS in determining the genetic diversity, p1 type, MLST profile and macrolide resistance of *M. pneumoniae* isolated between 2016 and 2024 in the UK. The data generated from the UK datasets were compared with genomic data from 290 *M. pneumoniae* strains isolated globally. The WGS approach provides more detailed information compared with traditional laboratory methods, indicating that WGS has potential application to support the genomic surveillance of *M. pneumoniae* and this approach can help in the detection of macrolide resistance in the circulating strains during outbreaks and seasonal epidemic surges. Further work is needed, using genomic epidemiological surveillance to understand epidemics within the population, especially with increasing antibiotic resistance.

## Supplementary Information

Supplementary Material 1.

## Data Availability

The sequence data for the 38 genomes of **M. pneumoniae** sequenced in this study were deposited in the SRA database (accession no. PRJNA1231462).

## Abbreviations

MLST: Multilocus sequence typing
MLVA: Multilocus variable-number tandem-repeat analysis
MRMP: Macrolide-resistant Mycoplasma pneumoniae
ST: Sequence type
WGS: Whole genome sequencing
SNP: Single nucleotide polymorphism
